# Porcine Intestinal Mucosal Peptides Target Macrophage-Modulated Inflammation and Alleviate Intestinal Homeostasis in Dextrose Sodium Sulfate-Induced Colitis in Mice

**DOI:** 10.3390/foods13010162

**Published:** 2024-01-03

**Authors:** Yucong Wang, Zhixin Xie, Xiaolong Wu, Lei Du, Zhengchen Chong, Rongxu Liu, Jianchun Han

**Affiliations:** 1 College of Food Science, Northeast Agricultural University, Harbin 150030, China; wangyucong1115@163.com (Y.W.); xiezhixin_1231@163.com (Z.X.); 18045605712@163.com (X.W.); dulei52i@163.com (L.D.); czcczcczc123123@163.com (Z.C.); 2 Heilongjiang Green Food Science Research Institute, Harbin 150030, China; rongxuliu@163.com

**Keywords:** inflammatory bowel disease, gut microbiota, peptide, anti-inflammation, RAW264.7 cell

## Abstract

Porcine intestinal mucosal proteins are novel animal proteins that contain large amounts of free amino acids and peptides. Although porcine intestinal mucosal proteins are widely used in animal nutrition, the peptide bioactivities of their enzymatic products are not yet fully understood. In the present study, we investigated the effect of porcine intestinal mucosal peptides (PIMP) on the RAW264.7 cell model of LPS-induced inflammation. The mRNA expression of inflammatory factors (interleukin 6, tumor necrosis factor-α, and interleukin-1β) and nitrous oxide levels were all measured by quantitative real-time PCR and cyclooxygenase-2 protein expression measured by Western blot. To investigate the modulating effect of PIMP and to establish a model of dextran sodium sulfate (DSS)-induced colitis in mice, we examined the effects of hematoxylin-eosin staining, myeloperoxidase levels, pro-inflammatory factor mRNA content, tight junction protein expression, and changes in intestinal flora. Nuclear factor κB pathway protein levels were also assessed by Western blot. PIMP has been shown in vitro to control inflammatory responses and prevent the activation of key associated signaling pathways. PIMP at doses of 100 and 400 mg/kg/day also alleviated intestinal inflammatory responses, reduced tissue damage caused by DSS, and improved intestinal barrier function. In addition, PIMP at 400 mg/kg/day successfully repaired the dysregulated gut microbiota and increased short-chain fatty acid levels. These findings suggest that PIMP may positively influence inflammatory responses and alleviate colitis. This study is the first to demonstrate the potential of PIMP as a functional food for the prevention and treatment of colitis.

## 1. Introduction

The term inflammatory bowel disease (IBD) refers to a group of chronic gastrointestinal disorders, the most common of which being Crohn’s disease and ulcerative colitis (UC). These disorders are distinguished by an abnormal immune system reaction and persistent intestinal inflammation. The consequences are distressing, marked by symptoms such as abdominal pain, persistent diarrhea, bloody stools, anemia, overwhelming fatigue, and unexplained weight loss. In recent years, several industrialized nations have displayed a worrisome trend in the incidence of IBD [1]. This shift, as highlighted by epidemiological studies, spotlighted North America and Northern Europe as the regions struggling with the highest rates of UC prevalence [2]. IBD is transforming into a global health concern and posing a threat to human well-being.

The complex pathophysiology of inflammatory bowel disease (IBD) includes immunological dysregulation, environmental variables, genetic predisposition, impaired intestinal barrier function, and disrupted gut microbiota balance [3,4]. It has been demonstrated that a disruption in the homeostasis of the gut microbiota is a key factor in the onset and development of IBD. The changed composition of the intestinal microbiota has a significant impact on immunological responses, physiological state, metabolic pathways, and intestinal homeostasis [5]. The immune system and inflammatory responses of the host can be influenced by this disturbance in intestinal microbiota homeostasis, either directly or indirectly, which can act as an initiator for the development of IBD-related diseases [6,7].

In recent years, selecting by-products with low biological value as raw materials to produce a range of bioactive substances with different functions has become a research trend in the food field [8]. Active peptides derived from food are natural protein fragments found in various food sources. These peptides possess significant biological activities and offer various health benefits. Typically resulting from the breakdown or enzymatic decomposition of larger protein molecules, these active peptides serve diverse physiological functions in the human body. The potential health benefits of food-derived active peptides, such as their anti-inflammatory, antibacterial, immunomodulatory, antioxidant, and antihypertensive properties, have aroused the interest of scientists. In mice with colitis caused by dextran sulfate sodium (DSS), the study showed that mulberry leaf protein hydrolysate could reduce inflammatory factors and tissue damage [9]. Furthermore, in adult zebrafish, the anti-inflammatory properties of clam peptide mmv2 were validated through its downregulation of the mRNA levels of genes linked to inflammation that were triggered by lipopolysaccharide (LPS) [10]. Moreover, a CTX-induced mouse model was established, and it was found that oyster peptide had a positive impact. It restored thymus, spleen, and liver functions, promoted cytokine secretion, and effectively ameliorated intestinal damage in mice [11]. Food-derived bioactive peptide is a new, safe, and efficient active substance with great potential for development [12]. At present, enzyme digestion and microbial fermentation are mainly used for the preparation of food-borne active peptides [13]. Although the microbial fermentation method has the advantages of low cost, wide source, and high yield. However, the fermentation and metabolism process are complicated and difficult to control, and it is not as good as the enzymatic method in terms of efficiency isolation and purification [13]. Enzymatic methods are more commonly used and have the advantages of low cost, high yield, safety, and specificity [14]. Therefore, enzymatic digestion has become the most important production method of bioactive peptides. Studies have shown that the enzymatic method can not only maintain the nutritional value of raw protein and release various bioactive peptides but also degrade the protein into peptides with different chain lengths, which is more favorable to be digested and absorbed by the body [15].

China, as the world’s largest producer of pork, accounts for about half of the world’s total pork production [16]. According to FAO, global pork production is about 125 million tons, with China’s contribution being 55.41 million tons, approximately 44.47% of the global total. Data reveal that over 80% of commercial pigs originate from foreign breeds. These pigs are bred by crossing one generation of imported foreign meat breeding pigs as the sire, followed by further crossings with imported foreign meat breeding pigs as the terminal sire, collectively referred to as the “ternary pig” [17]. “Ternary pigs” are favored for promotion due to their rapid growth, efficient feed utilization, high lean meat ratio, and notable economic benefits [18]. Given their prevalent market share and widespread use, we opted to select pig intestines from the “ternary pig” breed for our experiment. Furthermore, China holds the position of the world’s largest producer of natural pig sausage casings, responsible for around 80% of the world’s natural sausage casing production [19]. A substantial quantity of intestinal mucosa is generated during enteric coating processing. Porcine intestinal mucosa, the primary source material for extracting heparin, yields intestinal mucosa liquid in the production process. Research indicates that post-hydrolysis, porcine intestinal mucosa protein is abundant in not only protein, fat, fiber, and calcium, but also various amino acids, peptides, and nucleotides [20]. The finding showed that the addition of porcine intestinal mucosal proteins to the diet can significantly increase the daily weight gain of piglets, reduce weaning stress, and reduce mortality [21]. Furthermore, the addition of hydrolyzed porcine intestinal mucosal tissue to post-weaning feeds can replace up to 6% of fishmeal without detriment to performance [22]. Therefore, porcine intestinal mucosal proteins have nutritional properties such as high animal absorption and utilization and good palatability.

Currently, studies on porcine intestinal mucosa have only shown the immunomodulatory effects of mucin-o-glycans, whereas the enzymatic products of porcine intestinal mucosal proteins have been less studied [23]. Therefore, using the LPS-induced inflammation model of RAW264.7 cells and the DSS-induced colitis mouse model, respectively, as models of inflammation and intestinal microecology, porcine intestinal mucosal peptide (PIMP) was utilized as a raw material in this study in order to investigate the mechanisms of its regulatory effects on immune response and intestinal microecology. This research will provide evidence for the ability of PIMP to control the immune response and ameliorate IBD. It will also provide a scientific basis for the continued use of PIMP in food and medicine.

## 2. Materials and Methods

### 2.1. Preparation of Porcine Intestinal Mucosal Peptides (PIMP)

Fresh pig intestines were obtained from the “ternary pig” (Duroc × Lancashire × Yorkshire (DLY) pigs), purchased from the local market (Harbin, Heilongjiang, China). Following the method outlined by Li with minor adaptations [24]. After scraping out the inner wall mucosa, distilled water and mucosal quality were combined in a 1:3 (*w*/*v*) ratio. A homogenizer (T25, IKA, Staufen, Germany) was used to homogenize the mixture for two minutes at 5000 rpm. The homogenate solution is centrifuged at 8000 rpm for five minutes to obtain the supernatant, which is the porcine intestinal mucosa supernatant. PIMP was then prepared as described below. The homogenate solution was pre-heated for ten minutes in a DKZ-2C shaker (Haidian, China) set at 37 °C. Enzymatic hydrolysis was started by adjusting the pH with 1 M HCl to 3.0. Pepsin, which accounted for 1.0% of the sample, was then added and processed for two hours. Following completion, the mixture was inactivated for ten minutes in a bath of boiling water. The hydrolysate was allowed to come to room temperature before being centrifuged for 20 min at 8000 rpm (H1750R, Cence, China) in order to separate the supernatant. After passing the supernatant through a 0.45 μm filter, a 100 Da reverse osmosis membrane (BO-NA-GM-19, BoNa, Jinan, China) was used for desalination. After desalination, the hydrolysate was further processed through a Polyether sulfone membrane (5 kDa, Cobetter, Hangzhou, China) in an ultrafiltration device (iBio II, RJBIO, Suzhou, China). The final solution was vacuum freeze-drying (Alpha 1–2, CHRIST, Osterode, Germany), which was then kept until needed at −20 °C.

### 2.2. Sodium Dodecyl Sulfate-Polyacrylamide Gel Electrophoresis (SDS-PAGE)

The molecular weight of PIMP was determined by sodium dodecyl sulfate-polyacrylamide gel electrophoresis (SDS-PAGE) according to Laemmli [25]. The electrophoresis included a 10% separation gel and a 5% concentration gel. Samples (5 mg/mL) were diluted with an equal volume of loading buffer and boiled for 5 min. A multicolor pre-stained protein marker (Epizyme, WJ101, Shanghai, China) covering a protein range of 10–250 kDa was added to the gel. After electrophoresis, Coomassie Brilliant Blue R-250 (Solarbio, Beijing, China) was used for protein visualization. The gels were then destained and imaged using a gel imaging system (c500, Azure Biosystems, Dublin, CA, USA).

Tricine- SDS-PAGE was performed according to the established protocol [26]. Electrophoresis was performed using a 20% separating gel, 10% interlayer gel, and 4% concentrated gel. The samples (5 mg/mL) were diluted with an equal volume of loading buffer, and then boiled for 5 min. Pre-stained ultra-low-molecular-weight protein marker (Solarbio, Beijing, China) with the protein range of 3.3–31 kDa was added to the gel. After electrophoresis, the protein was visualized using Coomassie Brilliant Blue R-250. The gels were then destained and imaged using a gel imaging system (c500, Azure Biosystems, Dublin, CA, USA).

### 2.3. Cell Culture

Dulbecco’s Modified Eagle Medium (DMEM) was used to cultivate the RAW264.7 cell line, which was obtained from Procell Life Science & Technology in Wuhan, China. The medium was supplemented with 10% fetal bovine serum and 1% penicillin/streptomycin, both of which were obtained from the same source. The RAW264.7 cells were cultured at 37 °C with 5% CO_2_ in a sterile, humidified cell incubator.

### 2.4. Cell Viability Assay

Using the cell counting kit-8 (CCK8) technique, the effect of PIMP on the survival rate of RAW264.7 cells was evaluated [27]. During their logarithmic growth phase, RAW264.7 cells were seeded at a density of 1 × 10^5^ cells/mL in a 96-well plate. A total of 200 μL of cell suspension was added to each well as a supplement. Following a 24 h seeding period, the cells were treated with LPS (1 μg/mL) for one hour, and then PIMP (0, 5, 20, 80, 320, and 1280 μg/mL) for three hours. The supernatant was then collected. After the incubation period, each well was filled with 10 μL of CCK8 solution, and the cultures were left in the dark for an extra two hours. After that, a microplate reader was used to measure the samples’ absorbance at 450 nm.

### 2.5. Detection of Nitric Oxide (NO)

The method of NO production was followed by Noh [27]. During their logarithmic growth phase, RAW264.7 cells were seeded at a density of 1 × 10^5^ cells/mL in a 96-well plate. Following a 24 h incubation period, the cells were subjected to one hour of LPS (1 μg/mL) treatment, followed by exposure to PIMP cell high-dose (PIMPCH, 80 μg/mL) and low-dose (PIMPCL, 20 μg/mL). Then, the cell culture supernatants was performed utilizing an NO detection kit (Beyotime, Jiangsu, China), following the instructions provided by the manufacturer.

### 2.6. Experimental Animals

Male C57BL/6J mice, six weeks old, were kept in a controlled environment with a 12-h light/dark cycle, 24 ± 2 °C, and 50 ± 5% relative humidity (Beijing Vital River Biotechnology Co., Ltd., Beijing, China). Water and standard feed (AIN-93G, Nanjing Trophy Feed Technology Co., Ltd., Nanjing, China) were provided food ad libitum. The Northeast Agricultural University Animal Ethics Committee (NEAUEC20230426) gave its approval for this study’s ethical conduct. The National Institutes of Health Guide for the Care and Use of Laboratory Animals contained guidelines that the research followed. Strict procedures were followed to reduce any possible stress or discomfort the animals might have experienced during the experiment.

### 2.7. Experimental Design and Animal Grouping

An amount of 2.5% DSS (molecular weight 36,000–50,000, MP Biomedicals, Solon, OH, USA) was used to produce colitis in mice in order to evaluate the therapeutic effect of PIMP on colitis. After randomization, fifty mice were split up into five groups (n = 10). Here is the configuration of the experiment: (1) For 14 days, mice in the control group received 200 μL of PBS (pH 7.2) every day through gavage. (2) For seven days, the DSS group was given 200 μL of PBS (pH 7.2) every day through gavage. On day 8, they were given water with 2.5% DSS, and this continued for seven days until their euthanasia on day fifteen. (3) For 14 days, the positive control group received 200 μL of drinking water through gavage with 5-Aminosalicylic acid (5-ASA) (75 mg/kg) daily. (4) Mice in the PIMP low-dosage group (PIMPL) received a daily gavage dose of 100 mg/kg of PIMP for a period of 14 days. (5) For 14 days, a daily gavage dose of 400 mg/kg of PIMP was administered to the PIMP high-dose group (PIMPH) of mice. On day 1, the 5-ASA, PIMPL, and PIMPH groups were given regular water. On day 8, they were given 2.5% DSS in their drinking water, and on day 15, they were put to death. For analysis, mouse feces, serum, colon tissues, and cecal contents were collected and kept at −80 °C. The dosages of 100 mg/kg/day and 400 mg/kg/day, which were determined using the body surface area normalization method, are 1.23 g and 4.92 g, respectively, when converted to a human equivalent dose for a 60 kg individual [28].

### 2.8. Disease Activity Index (DAI) and Histological Evaluation

The mice were weighed every day at 9:00 in the morning during the trial. By dividing the difference between the daily weight variance and the weight from the day before by the weight measured prior to the start of the modeling, the amount of weight loss was calculated. Every day, the amount of blood in the stool and the viscosity of the feces were measured and recorded. An important metric for determining the degree of intestinal inflammation is the DAI. The degree of weight loss, the consistency of the stool, and the presence of blood in the stool were the three main variables that were evaluated to determine DAI [29]. For histological analysis, colon tissue samples were obtained from six randomly selected mice in each group. These samples were fixed in 4% paraformaldehyde overnight at 4 °C before being dehydrated in PBS with 30% sucrose. Alcian blue and hematoxylin eosin (H&E) staining were carried out in accordance with standard techniques [30]. The stained samples were examined under an Aperio Versa 8 microscope (Leica, Wetzlar, Germany) for histopathological analysis.

### 2.9. Myeloperoxidase (MPO) Activity Detection

Colon tissue samples were weighed. Physiological saline (100 μL per 10 mg of tissue) was added. After homogenizing the tissue, the supernatant was obtained by centrifuging the mixture for 10 min at 4 °C at 12,000 rpm. The recovered supernatant was subsequently introduced into a plate with 96 wells. To every well, 50 μL of the TM chromogenic solution was applied. For five minutes, the plate was incubated at room temperature. After adding the stop solution, a microplate reader was used to measure the OD at 450 nm.

### 2.10. Quantitative Real-Time Polymerase Chain Reaction (qRT-PCR)

The protocol was followed by the established method [31]. RAW264.7 cells were cultured with PIMP in the presence or absence of LPS (1 μg/mL). Using the TRIzol reagent, total RNA was extracted from the colon tissue and RAW264.7 cells in accordance with the guidelines provided in the manual. Using a Transcription cDNA Synthesis Kit, the extracted RNA was reverse-transcribed into complementary DNA (cDNA). Table S1 contains primer information. The CFX96^TM^ system (Bio-Rad, Hercules, CA, USA) in conjunction with the FastStart SYBR Green Master Mix (Takara SYBR^®^ Pre-mix Ex Taq^TM^ II, Dalian, China) was used to conduct the PCR reactions. A typical two-step protocol was used for the PCR amplification: 40 cycles of 95 °C for 3 s and 60 °C for 30 s were followed by one cycle at 95 °C for 30 s. The samples were subjected to melting curve analysis. The internal reference for relative expression was GAPDH mRNA.

### 2.11. Western Blot Analysis

The protocol was based on our previous laboratory experience [31]. The RAW264.7 cells were cultivated either without the addition of samples or in the presence of 1 μg/mL LPS or a control solution. Following the designated incubation period, cells were harvested and total homogenates were made using the previously outlined procedure [32]. The BCA protein assay kit was used to measure the protein concentration. Protein samples were separated using 10% and 12% composition sodium dodecyl sulfate-polyacrylamide gel electrophoresis (SDS-PAGE) gels. Post-separation, a wet transfer method was employed to transfer the proteins onto polyvinylidene fluoride membranes. The membranes were blocked by immersing them for an hour at room temperature in tris-buffered saline containing 5% skim milk and 0.5% Tween-20 (TBST). Subsequently, the membrane was incubated at 4 °C for an entire night with the primary antibody. The following were the antibodies and their dilutions: cyclooxygenase-2 (COX-2) (1:1000; Proteintech; Cat#66351-1-lg); Nuclear factor κB (NF-κB p65) (1:1000; Proteintech; Cat#10268-1-AP), p-NF-κB p65(Ser536) (1:1000; CST; Cat#3033); Inhibitor of NF-κB (IκB) (1:1000; Proteintech; Cat#10268-1-AP), p-IκB (Ser536) (1:1000; CST; Cat#3033); Claudin-1 (1:1000; Proteintech; Cat#13050-1-AP), Occludin (1:1000; Proteintech; Cat#27260-1-AP), zonula occludens-1 (ZO-1) (1:2000; Proteintech; Cat#21773-1-AP). Following incubation, membranes were incubated with secondary antibodies (1:2000, Proteintech, Cat#66031-1-lg, Cat#66009-1-lg) for 1 h at room temperature. They were then rinsed three times with TBST for 10 min each. Protein bands have been identified using the Amersham Imager 600 (General Electric Healthcare Life Sciences, Chicago, IL, USA) following three more TBST washes.

### 2.12. Short-Chain Fatty Acids (SCFAs)

With a few minor modifications, the Wang method was used to quantify SCFAs in mouse feces [33]. Gas chromatography (GC-2014C, Shimadzu Corporation, Kyoto, Japan) was used to examine the SCFA. After mixing 1 mL of 0.1% phosphoric acid solution with the 0.1 g mouse feces sample, centrifugation and vertexing were performed for 10 min at 4 °C and 15,000× *g*. By combining it in a 1:1 volume ratio with ethyl acetate, the supernatant was extracted. Following a 0.45 μm filter membrane filter, the ethyl acetate extraction phase was analyzed using a DB-WAX capillary column (30 m × 0.32 mm, 0.25 μm) in GC-MS. The carrier gas was high-purity helium flowing at a rate of 1.0 mL/min. A 1 μL injection volume was used, and the injection port temperature was kept constant at 220 °C. An electron impact ion source with an ion source temperature of 230 °C and an interface temperature of 220 °C were used in the mass spectrometry system.

### 2.13. Intestinal Microbiota

Fecal samples were obtained on the final day of a mouse colitis modeling research and stored in 1.5 mL sterile centrifuge tubes. After extracting the genomic DNA, the V3-V4 region was amplified using PCR (341F: 5′-ACTCCTACGGGAGGCAGCAG-3′, 806R: 5′-GGACTACHVGGGTWTCTAAT-3′). After being purified with Agencourt AMPure XP, the DNA fragments were labeled and libraries were built. The Illumina HiSeq 2500 platform was used for further sequencing, which produced raw data that were cleaned to produce clean data. Employing the divisive amplicon denoising algorithm, denoising was performed, yielding amplicon sequence variants and a featured table. These variants were then compared with established databases for taxonomic annotation, enabling diversity analysis and functional predictions based on operational taxonomic units (OTUs). The bioinformatics analysis was conducted on the Novagen Microbial Amplicon System.

### 2.14. Data Analysis

The data presented in this study, which came from at least three different and independent studies, are reported as the mean ± standard error of the mean (SEM). One-way analysis of variance (ANOVA) was used to evaluate the significance of differences between different groups [34]. Software named SPSS 20.0 was used to administer Duncan’s test and further evaluate the discrepancies. Statistical significance was defined as a *p*-value of less than 0.05 (*p* < 0.05).

## 3. Results

### 3.1. Molecular Weight

**Figure 1:**
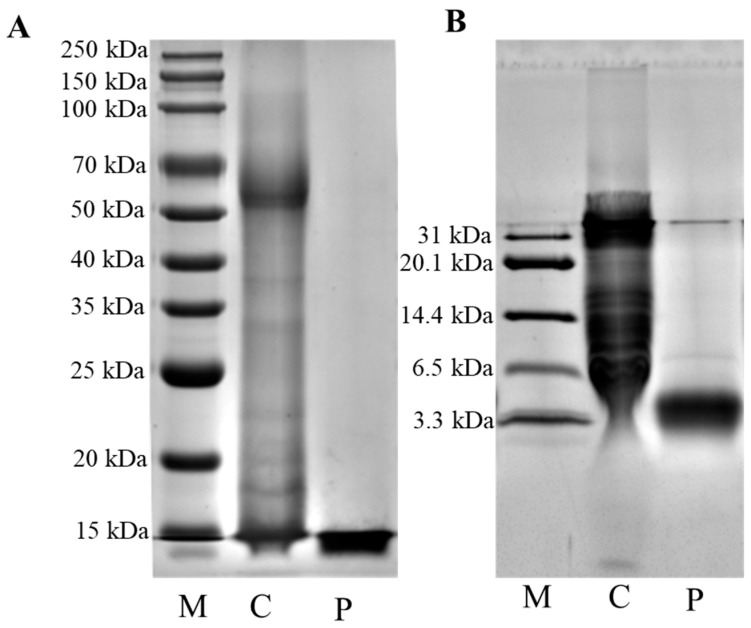
(**A**) SDS-PAGE image of porcine intestinal mucosa supernatant and porcine intestinal mucosal peptides. (**B**) Tricine SDS-PAGE image of porcine intestinal mucosal proteins and porcine intestinal mucosal peptides. M: marker; C: porcine intestinal mucosa supernatant; P: porcine intestinal mucosal peptides.

As shown in Figure 1, the supernatant derived from porcine intestinal mucosa was abundant in proteins, showcasing molecular weights ranging between 15–250 kDa, without distinct bands within the 3.3–31 kDa range. Conversely, upon the addition of pepsin, an enzymatic reaction ensued. The molecular weight of the enzymatically digested proteins significantly degraded to 3.3 kDa, displaying clear bands. This confirmed the transformation of proteins in the porcine intestinal mucosa supernatant into small-molecule peptides by pepsin, indicating the crucial role of pepsin in this process. Nonetheless, further investigation is required to delineate the structure of porcine intestinal mucosa peptides.

### 3.2. Effect of Porcine Intestinal Mucosal Peptide on Cell Viability of Lipopolysaccharide Induced Macrophages

**Figure 2:**
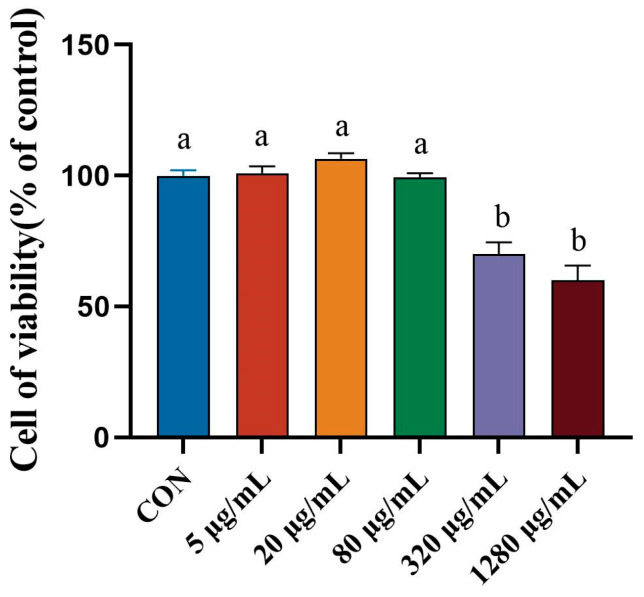
Effect with different concertation of PIMP on cytotoxicity of RAW264.7 cells. The results are shown as means ± SEM, and significant differences (*p* < 0.05) are identified between the various lowercase letters (*p* < 0.05).

To further study the anti-inflammatory mechanisms of PIMP, in vitro experiments have been conducted. The CCK8 test was used to determine how different PIMP doses affected the activity of RAW264.7 cells. A concentration-dependent effect was evident in Figure 2, where a rise in concentration resulted in a decrease in cell viability. When the concentration exceeds 80 μg/mL, a significant reduction in cell viability is observed (*p* < 0.05). For the following tests, concentrations of 20 μg/mL and 80 μg/mL were chosen.

### 3.3. Inhibition of Inflammatory Responses by Porcine Intestinal Mucosal Peptide

**Figure 3:**
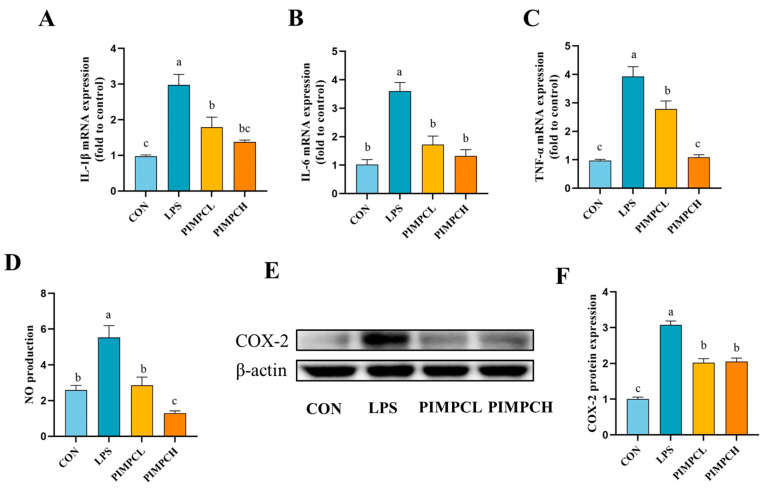
Impact of PIMP on inflammation in RAW264.7 cells stimulated by LPS. (**A**–**C**) qRT-PCR was used to identify the mRNA expression of TNF-α, IL-6, and IL-1β. (**D**) NO production. (**E**) Sample images from the COX-2 Western blot study. (**F**) Relative COX-2 protein expressions. The data are shown as means ± SEM, showing significant differences (*p* < 0.05) across the various lowercase letters. Control group (CON): cell with untreated; LPS group (LPS): cell with treated lipopolysaccharide (1 μg/mL); PIMPCL: cell with low dose (20 μg/mL) of porcine intestinal mucosal peptide and lipopolysaccharide (1 μg/mL); PIMPCH, cell with high dose (80 μg/mL) of porcine intestinal mucosal peptide and lipopolysaccharide (1 μg/mL);.

The group treated with PIMP significantly suppressed the high levels of pro-inflammatory cytokines caused by LPS, such as interleukin-1β (IL-1β), interleukin 6 (IL-6), and tumor necrosis factor-α (TNF-α), according to qRT-PCR analysis (Figure 3A–C). In addition, the effects of PIMP on NO level (Figure 3D) and COX-2 (Figure 3E,F) protein expression in these cells were also evaluated. The NO test results verified the significant decrease achieved by the PIMP group (*p* < 0.05). Western blot was used to measure COX-2 protein expression in order to better investigate the molecular process. Our findings showed that PIMP treatment effectively inhibits LPS-induced upregulation of COX-2.

### 3.4. Porcine Intestinal Mucosal Peptide Inhibits Macrophage Inflammatory Response Induced by Lipopolysaccharide Via the NF-κB Signaling Pathway

**Figure 4:**
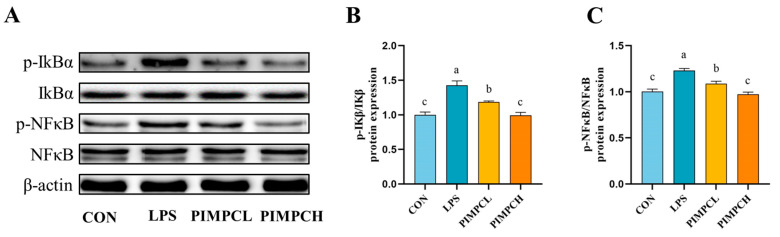
Effect of PIMP on the inflammation in LPS-induced RAW264.7 cells. (**A**) Representative images of Western blotting analysis of IκBα, p-IκBα, NF-κB, and p-NF-κB. (**B**,**C**) Relative protein expressions of IκBα, p-IκBα, NF-κB, and p-NF-κB. The values are presented as means ± SEM, with significant differences in different lowercase letters (*p* < 0.05). Control group (CON): cell with untreated; LPS group (LPS): cell with treated lipopolysaccharide (1 μg/mL); PIMPCL: cell with low dose (20 μg/mL) of porcine intestinal mucosal peptide and lipopolysaccharide (1 μg/mL); PIMPCH, cell with high dose (80 μg/mL) of porcine intestinal mucosal peptide and lipopolysaccharide (1 μg/mL).

Western blot analysis was used to examine the effects of PIMP on the NF-κB signaling pathway (Figure 4). IκBα and NF-κB phosphorylation significantly increased in the LPS group, suggesting an active inflammatory response (*p* < 0.05). Nevertheless, the application of PIMP resulted in a significant enhancement of phosphorylated protein expression levels (*p* < 0.05). These findings also show that PIMP has a strong ability to block the NF-κB signaling pathway from being activated.

### 3.5. Porcine Intestinal Mucosal Peptide Alleviates Symptoms of Acute Colitis in Mice

**Figure 5:**
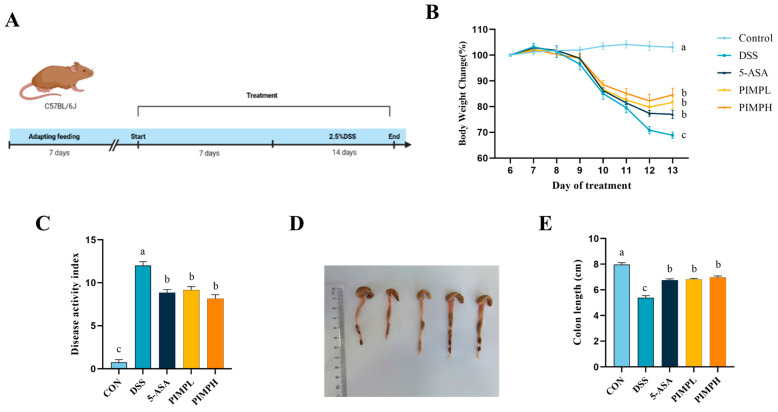
Effects of PIMP on DSS-induced colitis in mice. (**A**) Experimental design: mice were categorized into five groups: CON (untreated control), DSS (DSS only), PIMPL (DSS + 100 mg/kg/d PIMP treatment), PIMPH (DSS + 400 mg/kg/d PIMP treatment), and 5-ASA (DSS + 75 mg/kg/d 5-ASA treatment). (**B**) Changes in body weight and (**C**) disease activity index (DAI) score: graphs showing alterations in body weight and DAI score during the representative DSS treatment. (**D**) Colon images and (**E**) measurements of colon length in mice from each group. The values are presented as means ± SEM, with significant differences in different lowercase letters (*p* < 0.05). Control group (CON): non-DSS-induced; DSS group (DSS): with 2.5% DSS induced for 14 days; 5-ASA group (5-ASA): 5-ASA (75 mg/kg/d) for 14 days and 2.5% DSS for the last 7 days; PIMPL group (PIMPL): porcine intestinal mucosal peptide with low-dose group (100 mg/kg/d) for 14 days and 2.5% DSS for the last 7 days; PIMPH group (PIMPH): porcine intestinal mucosal peptide with high-dose group (400 mg/kg/d) for 14 days and 2.5% DSS for the last 7 days.

Using a DSS-induced colitis mouse model, we administered a 2.5% DSS solution for 7 days in order to evaluate the protective effectiveness of PIMP against colitis (Figure 5A). We monitored daily changes in body weight and DAI scores during the DSS therapy period. The body weight of the DSS-exposed mice was significantly lower than that of the control group. This weight loss was decreased by PIMP supplementation (Figure 5B) (*p* < 0.05). The DSS group outscored the 5-ASA group (8.86 ± 0.34) and the PIMP group (*p* < 0.05) in terms of DAI scores, whereas there was not a significant distinction between the PIMPL group (9.14 ± 0.4) and the PIMPH group (8.14 ± 0.46) (*p* > 0.05).

The colon length of the mice in the DSS group (5.38 ± 0.16 cm) was significantly less than that of the control group (7.97 ± 0.13 cm) (*p* < 0.05), as shown in Figure 5D,E. In comparison to the DSS group, the colon shortening was lessened by the colon lengths in the PIMP group (6.82 ± 0.07 cm & 6.97 ± 0.11 cm) and 5-ASA group (6.75 ± 0.09 cm). When everything is considered, these results indicate that PIMP significantly decreased the severity of colitis (*p* < 0.05).

### 3.6. Porcine Intestinal Mucosal Peptide Alleviates Tissue Damage in Dextrose Sodium Sulfate Induced Colitis

**Figure 6:**
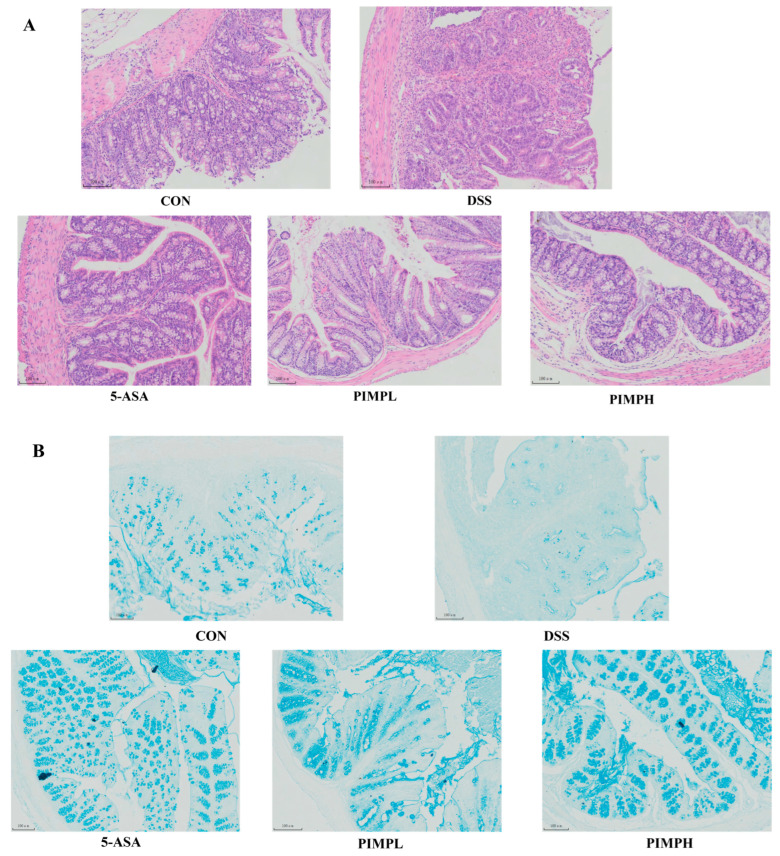
Impact of PIMP on histopathological injury and mucosal layer in DSS-induced colitis. (**A**) Histopathological analysis: representative images of colon sections stained with H&E for each experimental group are shown (magnification, 400×). Colon tissue injury was characterized by inflammatory cell infiltrations, as indicated by arrows in the images. (**B**) Mucosal layer examination: representative images of colon sections stained with Alcian blue for each group are presented (magnification, 400×). Control group (CON): non-DSS-induced; DSS group (DSS): with 2.5% DSS induced for 14 days; 5-ASA group (5-ASA): 5-ASA (75 mg/kg/d) for 14 days and 2.5% DSS for the last 7 days; PIMPL group (PIMPL): porcine intestinal mucosal peptide with low-dose group (100 mg/kg/d) for 14 days and 2.5% DSS for the last 7 days; PIMPH group (PIMPH): porcine intestinal mucosal peptide with high-dose group (400 mg/kg/d) for 14 days and 2.5% DSS for the last 7 days.

The results presented in Figure 6A demonstrate the structural integrity of colon epithelial cells and mucosal crypts in the control group, characterized by their structure integrity and the absence of inflammatory cell infiltration. On the other hand, the DSS group has significant crypt structural damage, incomplete mucosal epithelium, noticeable mucosal damage, worsened edema, and increased inflammatory cell infiltration. In contrast to the DSS group, the 5-ASA group and the PIMP group exhibit definite improvements in inflammatory cell infiltration and mucosal injury (*p* < 0.05). There is an obvious increase in the protective effect in the PIMPH group. This is evidenced by a significant improvement in crypt structures and a more complete epithelial structure, demonstrating the profound impact of PIMP on reducing damage and inflammation in the colon.

Mucus secreted by goblet cells is essential for protecting the intestinal epithelial cells. Using a stain made especially for mucins, Alcian blue, we assessed the integrity of the mucus layer. When compared to the control group, the data displayed in Figure 6B demonstrate a significant decrease in the quantity of goblet cells and irregular size in the DSS group, indicating the disruption of the intestinal mucosal layer structure caused by DSS-induced colitis. Interestingly, a positive shift was noticed with the implementation of PIMP. Goblet cells increased in number and exhibited an organized arrangement. This finding suggests that PIMP has the potential to regulate goblet cells effectively in DSS-induced colitis, thereby reducing the damage to the intestinal mucosa.

### 3.7. Porcine Intestinal Mucosal Peptide Restores Intestinal Barrier Damage in Dextrose Sodium Sulfate Induced Colitis Mice

**Figure 7:**
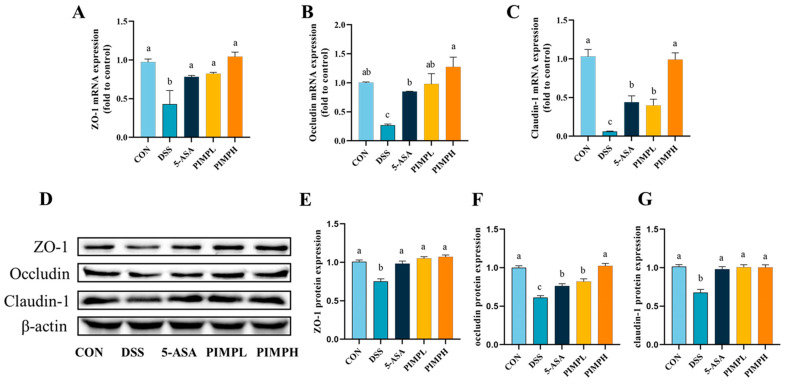
The impact of PIMP on the integrity of the intestinal barrier in mice with DSS-induced colitis. (**A**–**C**) mRNA expression levels of key proteins (ZO-1, Occludin, and Claudin-1) in the colon, determined by qRT-PCR. (**D**) Representative images from the Western blot analysis on ZO-1, Occludin, and Claudin-1 in the colon. (**E**–**G**) Relative protein expressions of ZO-1, Occludin, and Claudin-1. The values are presented as means ± SEM, with significant differences in different lowercase letters (*p* < 0.05). Control group (CON): non-DSS-induced; DSS group (DSS): with 2.5% DSS induced for 14 days; 5-ASA group (5-ASA): 5-ASA (75 mg/kg/d) for 14 days and 2.5% DSS for the last 7 days; PIMPL group (PIMPL): porcine intestinal mucosal peptide with low-dose group (100 mg/kg/d) for 14 days and 2.5% DSS for the last 7 days; PIMPH group (PIMPH): porcine intestinal mucosal peptide with high-dose group (400 mg/kg/d) for 14 days and 2.5% DSS for the last 7 days.

For the body to remain in a state of homeostasis, the intestinal barrier must remain intact. Disruptions in this barrier, such as those induced by DSS, can increase intestinal permeability. In order to maintain homeostasis between the intestinal environment and its external surroundings, tight junction (TJ) proteins, significant regulators of this permeability, are essential. ZO-1, Occludin-1, and Claudin-1, three important TJ proteins, were evaluated for mRNA and protein expression in order to evaluate the regulatory effect of PIMP on DSS-induced intestinal barrier disruption. As demonstrated in Figure 7, animals in the DSS group had mRNA levels of these TJ proteins in the colon that were significantly lower than those in the control group (*p* < 0.05). Notably, ZO-1 and Occludin-1 were not significantly different in the PIMPH group compared to PIMPL, whereas Claudin-1 expression was highly significant (*p* < 0.05). Furthermore, a statistically significant increase in Occludin-1 expression was observed in the PIMPH group relative to the 5-ASA group, indicating that PIMPH enhanced the intestinal barrier. Moreover, we used the Western blot technique to identify modifications in the tight junction proteins’ protein content. As shown, there was a significant (*p* < 0.05) downregulation of TJ protein expression in the DSS group when compared to the control group. But this tendency was reversed when 5-ASA and PIMP were added. In particular, ZO-1 and Claudin-1 did not show significance (*p* > 0.05), whereas Occludin-1 expression in the PIMPH group was significantly greater than that in the PIMPL group (*p* < 0.05). Therefore, PIMP effectively counteracted the DSS-induced downregulation of TJ protein expression.

### 3.8. Porcine Intestinal Mucosal Peptide Suppress Inflammatory Response in Dextrose Sodium Sulfate Induced Colitis

**Figure 8:**
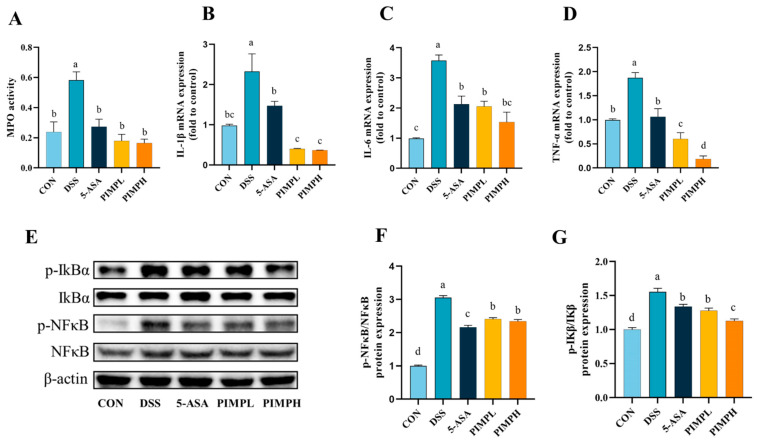
Impact of PIMP on inflammation in DSS-induced colitis mice. (**A**) Colon MPO activity: measurement of MPO activity in the colon tissue. (**B**–**D**) Gene expression analysis: mRNA levels of pro-inflammatory cytokines IL-1β, IL-6, and TNF-α in the colon. (**E**) Representative Western blot images showing the levels of IκBα, p- IκBα, NF-κBα, and p-NF-κB in the colon tissue. (**F**) Quantification of protein expressions, represented as the ratio of NF-κB, and p-NF-κB. (**G**) Quantification of protein expressions, represented as the ratio of IκBα and p-IκBα. The values are presented as means ± SEM, with significant differences in different lowercase letters (*p* < 0.05). Control group (CON): non-DSS-induced; DSS group (DSS): with 2.5% DSS induced for 14 days; 5-ASA group (5-ASA): 5-ASA (75 mg/kg/d) for 14 days and 2.5% DSS for the last 7 days; PIMPL group (PIMPL): porcine intestinal mucosal peptide with low-dose group (100 mg/kg/d) for 14 days and 2.5% DSS for the last 7 days; PIMPH group (PIMPH): porcine intestinal mucosal peptide with high-dose group (400 mg/kg/d) for 14 days and 2.5% DSS for the last 7 days.

Myeloperoxidase (MPO) is an enzyme that is widely distributed in macrophages and granulocytes and is essential to the immune system. As anticipated, the DSS group had a significant three-fold increase in MPO activity in comparison to the control group (Figure 8A) (*p* < 0.05). On the other hand, MPO activity was significantly reduced upon administration of PIMP or 5-ASA, successfully reducing the DSS-induced elevation in MPO levels (*p* < 0.05). It is well known that the production of inflammatory factors may trigger an immunological response and harm the mucosa of the intestine. Pro-inflammatory factors such IL-1β, IL-6, and TNF-α were examined and their mRNA expression evaluated in or-der to explore the regulatory potential of PIMP in mitigating DSS-induced inflammation (Figure 8B–D). The findings showed that the DSS group had significantly more pro-inflammatory elements than the CON group. On the other hand, eating 5-ASA or PIMP significantly decreased the expression of mRNA. In particular, compared to the 5-ASA group, both PIMP groups significantly reduced the expression of inflammatory IL-1β and TNF-α (*p* < 0.05). Furthermore, the PIMPH group demonstrated significantly reduced TNF-α expression in comparison to the PIMPL group, but the other two inflammatory factors did not demonstrate any relevance. To further study the mechanism of action of PIMP on the DSS-induced colitis model, a Western blot was performed to investigate the activation of key proteins within the IκB/NF-κB signaling pathway (Figure 8E–G). The results demonstrated that both PIMP and 5-ASA significantly inhibited DSS-induced NF-κB and IκB phosphorylation. Notably, PIMPH more significantly downregulated the protein expression of IκB compared to PIMPL. In conclusion, PIMP effectively ameliorated the inflammatory response in DSS-induced colitis.

### 3.9. Porcine Intestinal Mucosal Peptide Adjusts Short-Chain Fatty Acids (SCFAs) in Dextrose Sodium Sulfate Induced Colitis

**Figure 9:**
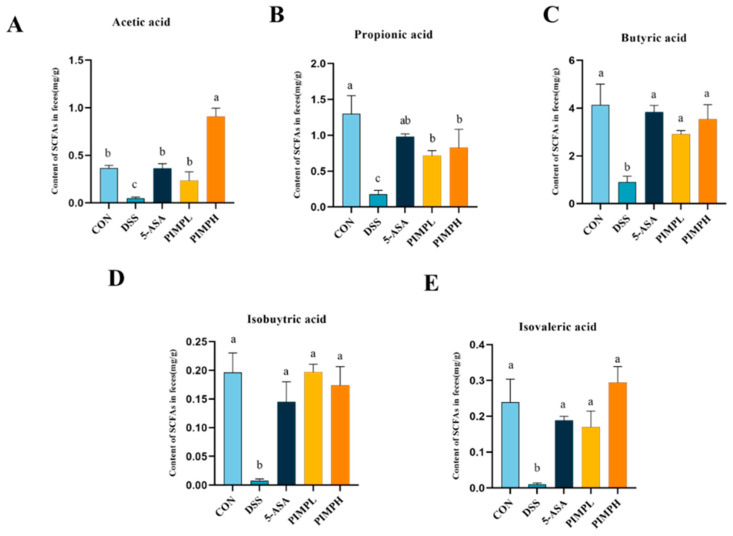
Impact of PIMP on SCFA production in the feces of mice with DSS-induced colitis. The levels of various SCFAs, including (**A**) acetic acid, (**B**) propionic acid, (**C**) butyric acid, (**D**) isobutyric acid, and (**E**) isovaleric acid, were assessed. The values are presented as means ± SEM, with significant differences in different lowercase letters (*p* < 0.05). Control group (CON): non-DSS-induced; DSS group (DSS): with 2.5% DSS induced for 14 days; 5-ASA group (5-ASA): 5-ASA (75 mg/kg/d) for 14 days and 2.5% DSS for the last 7 days; PIMPL group (PIMPL): porcine intestinal mucosal peptide with low-dose group (100 mg/kg/d) for 14 days and 2.5% DSS for the last 7 days; PIMPH group (PIMPH): porcine intestinal mucosal peptide with high-dose group (400 mg/kg/d) for 14 days and 2.5% DSS for the last 7 days.

Given the possible advantages these peptides may provide, the effect of PIMP on SCFAs in DSS-induced colitis is a topic of great interest (*p* < 0.05). SCFAs, produced through microbial fermentation, play a crucial role in regulating cytokines, exerting anti-inflammatory effects, and maintaining colon homeostasis, as supported by various studies [35]. Additionally, SCFAs positively influence the intestinal barrier and are the main source of energy for colon cells, which has an impact on diseases including non-alcoholic fatty liver disease, type 1 diabetes, and IBD [36]. Acetic acid, propionic acid, butyric acid, valeric acid, isobutyric acid, and isovaleric acid were the important SCFA concentrations that were significantly lower in the colon of mice with DSS-induced colitis than in the control group (*p* < 0.05) (Figure 9). In contrast to the DSS group, the 5-ASA group and the PIMP group both successfully raised these SCFA levels. What was very impressive was the strong impact that the PIMPH group displayed. This group exhibited a significantly more pronounced enhancement in SCFA content, indicating the potential of PIMP in positively modulating SCFAs in colitis (*p* < 0.05).

### 3.10. Porcine Intestinal Mucosal Peptide Regulates the Intestinal Microbiota in Mice with Dextrose Sodium Sulfate Induced Colitis

**Figure 10:**
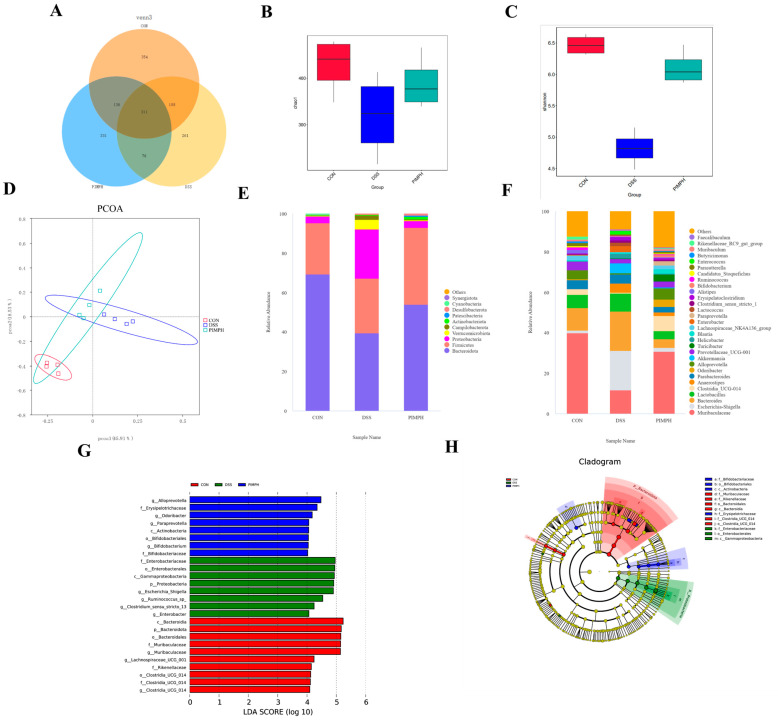
Impact of PIMP on the gut microbiome compositions in DSS-induced colitis mice. Microbiome sequencing data were collected from the control group (CON), DSS-treated group (DSS), and DSS-treated group with 400 mg/kg/d PIMP (PIMPH). (**A**) Venn diagrams indicated the unique and shared gut microbiota among the groups, determined by operational taxonomic unit (OTU) values. (**B**) Chao1 index and (**C**) Shannon index show the variation in species richness and evenness within the groups. (**D**) PCoA of the dissimilarities in microbial composition of each group at (**E**) phylum level and (**F**) genus levels. (**G**) Distribution histogram based on linear discriminant analysis (LDA). (**H**) Linear discriminant analysis effect size (LEfSe) analysis cluster tree. Control group (CON): non-DSS-induced; DSS group (DSS): with 2.5% DSS induced for 14 days; PIMPH group (PIMPH): porcine intestinal mucosal peptide with high-dose group (400 mg/kg/d) for 14 days and 2.5% DSS for the last 7 days.

One of the main causes of intestinal inflammation is a disturbance of the homeostasis of the gut microbiota. Here, the effect of PIMPH on intestinal microbiota was examined using high-throughput sequencing of 16s rDNA. Alpha diversity is used to evaluate the diversity within the intestinal flora and mainly includes two indicators. The Chao1 score represents microbial abundance, whereas the Shannon index represents microbiota diversity (Figure 10B,C). The DSS group demonstrated a statistically significant decrease in both the Chao1 index and Shannon index (*p* < 0.05) when compared to the control group. It is interesting to note that PIMPH or 5-ASA treatment reversed the colitis-induced decline in species abundance. The chao1 index rose in the 5-ASA and PIMPH groups compared to the DSS group, although the change was not statistically significant (*p* > 0.05). These results demonstrate that the variety and richness of the gut microbiota in mice were not significantly changed by DSS alone. However, the variety and richness of the gut microbiota were significantly increased by the PIMPH supplementation.

β-diversity analysis serves as a crucial tool for understanding the variation in species composition among different groups. Consequently, our study found significant differences in the microbiota composition between the DSS group and the control group based on principal coordinate analysis (PCoA) (Figure 10D). The makeup of the microbiota changed with the introduction of PIMP. Nevertheless, no significant differences were produced by this modification (*p* > 0.05).

Figure 10E displays the gut microbiota composition of each group at the phylum level. The findings indicated that the majority of the bacteria in the normal control group were *Firmicutes* and *Bacteroidetes*. Our findings demonstrated a substantial decrease in the relative abundance of *Firmicutes* and *Bacteroidetes* in the DSS group as compared to the control group (*p* < 0.05). The relative abundance of *Verrucomicrobia* and Proteobacteria increased significantly. Comparing the DSS group with PIMPH group, significant shifts were observed. *Proteobacteria* and *Wartus* displayed a significant drop (*p* < 0.05) in relative abundance, but *Firmicutes*, *Bacteroidetes*, and *Campylobacterota* exhibited a significant increase. *Verrucomicrobia*’s relative abundance was also greatly decreased. These findings imply that PIMPH is important at the phylum level in reestablishing the species composition in colitis caused by DSS (*p* < 0.05).

The impact of PIMPH on the intestinal microbiota of DSS mice was examined in further detail in the following analysis, with a particular emphasis on genus level analysis. As seen in Figure 10F, the DSS mice demonstrated a significant drop in *Muribaculaceae* (*p* < 0.05) along with a large increase in *Escheri-Shigella* relative abundance when compared to the control group. However, when exposed to PIMPH, the DSS mice displayed a significant shift. Specifically, the relative abundance of *Muribaculaceae* and *Lachnospira* experienced a significant increase, while *Escheri-Shigella* showed a significant decline (*p* < 0.05).

The above findings highlighted the positive impact of PIMPH on enhancing the diversity and microbial composition of intestinal microbiota in DSS-induced colitis (Figure 10G,H). Utilizing LefSe analysis with an LDA score threshold of 4.0, different groups of dominant taxa were identified. In the control group, the prevailing bacteria were *Bacteroidia*, *Bacteroidota*, and *Bacteroidales*. DSS group exhibited an enrichment of *Enterobacteriaceae*, *Enterobacterales*, *Escherichia_Shigella*, and similar taxa. The PIMPH group displayed an enrichment of dominant species such as *Alloprevotella*, *Odoribacter*, and *Bifidobacteriales*.

## 4. Discussion

Targeted medication therapy is the current treatment for inflammatory bowel disease (IBD), a chronic, non-specific inflammatory illness of the intestines [37]. However, it is limited by the risk of intolerance and adverse effects. The introduction of novel raw materials is urgently needed in order to produce new, effective medications for the prevention or treatment of inflammatory bowel disease (IBD) because of the lengthy treatment duration, restricted therapeutic efficacy, high cost, and adverse effects of current medications [38,39]. Comprehensive utilization of agro-animal processing industry by-products can be more efficient as they contain a large number of nutrients and functional factors [40]. The ‘ternary pig’ holds a substantial market share due to its notable advantages such as rapid growth, high disease resistance, robust vitality, efficient feed conversion ratio, and a high proportion of lean meat. Hence, we opted to use this breed as the source of pig intestines for our experiment. The results of the study demonstrated that porcine intestinal mucosal peptide (PIMP) was able to successfully stop weight loss, lessen tissue damage, control the expression of TJ proteins, manage the stability of intestinal microecology in mice, and control the amount of colonic inflammatory components. Furthermore, through NF-κB signaling, PIMP suppressed the content of pro-inflammatory cytokines. These factors confirmed that PIMP could improve colitis disease.

Macrophages are the main component of the mononuclear phagocyte system. An important part of the innate immune response is played by the macrophage-based intrinsic immune system [41]. Lipopolysaccharide (LPS) is one of the most common and harmful inflammatory factors [42]. Research indicates that bioactive peptides derived from food, like rice glutathione, sturgeon peptide, and European eel bioactive peptide, effectively suppress inflammatory cytokines in LPS-stimulated RAW264.7 macrophages, thereby minimizing the inflammatory response. These outcomes agree with the findings of our experiment [43,44,45].

DSS is known to elevate intestinal mucosal permeability, upregulate specific inflammatory factors, and disrupt the balance of intestinal microbiota, triggering inflammation [46]. Numerous pro- and anti-inflammatory cytokines are important participants in intestinal inflammation [47]. TNF-α, IL-1β, and IL-6 work together to stimulate the production of adhesion molecules, chemokines, and other cytokines, which in turn promotes inflammation [48]. Moreover, MPO is thought to be a sign of tissue injury and neutrophil infiltration, which destroys mucosa and activates an inflammatory response [49]. The outcomes shown that PIMP could downregulate mRNA levels and dramatically suppress the levels of MPO and pro-inflammatory cytokines TNF-α, IL-1β, and IL-6. These findings may indicate that PIMP has the capacity to control the inflammatory response in mouse colitis.

Excessive inflammatory responses and intestinal epithelial barrier disruption have been increasingly implicated in recent research as underlying causes of UC. The integrity of the epithelial barrier is crucial in protecting intestinal tissues from pro-inflammatory agents, preventing their entry into the lamina propria [50,51]. Central to this defense is TJ proteins, which serve as key components of the integrity of the epithelial barrier. Research has repeatedly shown that the intestinal epithelial barrier is compromised in ulcerative colitis (UC) when ZO-1, Claudin-1, and Occludin, essential TJ proteins, are not expressed normally [52,53]. Noteworthy advancements in this field include the observation that the neuropeptide vasoactive intestinal peptide can safeguard the intestinal epithelial barrier by modulating the redistribution of TJ proteins induced by bacteria [54]. Our experimental results have shown that PIMP can enhance the mRNA and protein expression of TJ proteins.

New research indicates that disturbed microbiota contributes to the emergence of UC. Recent clinical findings have established a strong connection between UC and disruption in microbiota homeostasis, indicating that inflammatory response might be linked to these disruptions [55]. Given the significant influence of intestinal microbiota on regulating host immunity, disruptions could lead to dysregulated immune responses, potentially triggering UC. In our study, we discovered that PIMP effectively counteracted the decrease in SCFAs induced by DSS. Moreover, mice consuming high doses of PIMP exhibited potentially increased SCFA production by intestinal microbiota in their colon. This suggests a potential enrichment of specific SCFA-producing bacterial groups. As a result, we concentrated our research on the microbiota in the digestive tract of mice given large PIMP dosages.

Our findings revealed a reduction in both the chao1 index and Shannon index, indicating decreased microbial diversity in the DSS-exposed group. The result of PCoA showed changes in the composition of the gut flora. *Proteobacteria*, such as *Escherichia coli-Shigella*, have been found to significantly increase at the phylum level. This is a distinctive trait linked to both DSS-induced colitis in mice and IBD [56]. *Proteobacteria* are Gram-negative bacteria containing LPS in the outer membrane, highlighting their ecological and pathogenic significance [57]. Microbial diversity and composition were restored as a result of the efficient counteraction provided by PIMP. *Proteobacteria* were found to be significantly less abundant, which is consistent with earlier research that linked high *proteobacteria* levels to IBD. *Firmicutes* and *Bacteroidetes*, prominent components of the intestinal microbiota, are known for their protective effects against IBD [58,59]. The relative abundance of *Bacteroidetes* and *Firmicutes* was partially regulated by PIMP treatment, which further supported the significance of their balance. This control was crucial in reversing the trend of weight loss seen in colitis produced by DSS, suggesting that PIMP may be useful as a therapy in the management of symptoms related to IBD. At the genus level, DSS exposure led to significant increase in *Escheri-Shigella*, a trend that was significantly attenuated by PIMP treatment. Additionally, the PIMP group exhibited higher relative abundances of beneficial taxa such as *Muribaculaceae* and *Lachnospira*. *Muribaculaceae*, belonging to the *Bacteroidetes* phylum, has been linked to enhanced longevity in mice [60,61]. In contrast, *Lachnospiraceae*, a member of the *Firmicutes* phylum, is typically found in low abundance in patients with colitis [62]. Our findings show that PIMP has a species-specific modulatory effect, highlighting its potential to support a more favorable composition of the gut microbiota in DSS-induced mice.

## 5. Conclusions

The results of this research highlight the possible use of PIMP in reducing intestinal microecology and inflammation associated with IBD. According to our research, PIMP decreased NO content and COX-2 protein expression while also reducing pro-inflammatory cytokines (IL-1β, IL-6, and TNF-α) in LPS-induced RAW264.7 cells. Additionally, PIMP reduced intestinal tissue damage, elevated tight junction protein content, inhibited pro-inflammatory factors, and regulated intestinal flora in an animal model of colitis. Overall, PIMP significantly improved intestinal microecology and reduced inflammatory responses in the intestines via modifying the IκB/NF-κB signaling pathway. Hence, the potential of PIMP as a bioactive compound derived from food and as a functional food for treating colitis warrants further exploration through clinical trials in the future.

## Data Availability

Data is contained within the article or Supplementary Material.

## Supplementary Materials

The following supporting information can be downloaded at: https://www.mdpi.com/article/10.3390/foods13010162/s1, Table S1. Primers of RT-qPCR.
