# Supplementary material for: Porcine Intestinal Mucosal Peptides Target Macrophage-Modulated Inflammation and Alleviate Intestinal Homeostasis in Dextrose Sodium Sulfate-Induced Colitis in Mice
Source: Foods. 2024 Jan 3;13(1):162. doi: 10.3390/foods13010162 (PMC10778919; doi:10.3390/foods13010162)
Supplement: Supplementary file 1 [file foods-13-00162-s001.zip › foods-2785367-supplementary.pdf]

Supplementary Table S1. Primers of RT-qPCR.

Gene	Forward Primer	Reverse Primer
IL-1β	TGACGGACCCCAAAAGATGA	TCTCCACAGCCACAATGAGT
IL-6	GAGGATACCACTCCCAACAGACC	AAGTGCATCATCGTTGTTCATACA
TNF-α	CCCTCACACTCAGATCATCTTCT	CTACGACGTGGGCTACAG
ZO-1	TCTTCCATCATTTCGCTGTGT	TCTGAAACCATCAAGTCCACA
Occludin	TGCGGTGACTTCTCCAAACT	GGGGAACGTGGCCGATAT
Claudin-1	TAATTGGCATCCTGCTGGGG	CTGGCCAAATTCATACCTGGC
GAPDH	TGGAGAAACCTGCCAAGTATGA	TGGAAGAATGGGAGTTGCTGT
